# High-resolution double vision of the allosteric phosphatase PTP1B

**DOI:** 10.1107/S2053230X23010749

**Published:** 2024-01-01

**Authors:** Shivani Sharma, Tamar Skaist Mehlman, Reddy Sudheer Sagabala, Benoit Boivin, Daniel A. Keedy

**Affiliations:** a Structural Biology Initiative, CUNY Advanced Science Research Center, New York, NY 10031, USA; b PhD Program in Biology, CUNY Graduate Center, New York, NY 10016, USA; c Department of Nanobioscience, College of Nanoscale Science and Engineering, SUNY Polytechnic Institute, Albany, NY 12203, USA; d Department of Chemistry and Biochemistry, City College of New York, New York, NY 10031, USA; e PhD Programs in Biochemistry, Biology and Chemistry, CUNY Graduate Center, New York, NY 10016, USA; University of Michigan, USA

**Keywords:** protein tyrosine phosphatase 1B, allosteric regulation, conformational heterogeneity, high-resolution crystallography

## Abstract

Protein tyrosine phosphatase 1B (PTP1B) plays important roles in cellular homeostasis and is a highly validated therapeutic target for multiple human ailments, including diabetes, obesity and breast cancer. However, much remains to be learned about how conformational changes may convey information through the structure of PTP1B to enable allosteric regulation by ligands or functional responses to mutations. High-resolution X-ray crystallography can offer unique windows into protein conformational ensembles, but comparison of even high-resolution structures is often complicated by differences between data sets, including non-isomorphism. Here, the highest resolution crystal structure of apo wild-type (WT) PTP1B to date is presented out of a total of ∼350 PTP1B structures in the PDB. This structure is in a crystal form that is rare for PTP1B, with two unique copies of the protein that exhibit distinct patterns of conformational heterogeneity, allowing a controlled comparison of local disorder across the two chains within the same asymmetric unit. The conformational differences between these chains are interrogated in the apo structure and between several recently reported high-resolution ligand-bound structures. Electron-density maps in a high-resolution structure of a recently reported activating double mutant are also examined, and unmodeled alternate conformations in the mutant structure are discovered that coincide with regions of enhanced conformational heterogeneity in the new WT structure. These results validate the notion that these mutations operate by enhancing local dynamics, and suggest a latent susceptibility to such changes in the WT enzyme. Together, these new data and analysis provide a detailed view of the conformational ensemble of PTP1B and highlight the utility of high-resolution crystallography for elucidating conformational heterogeneity with potential relevance for function.

## Abstract 2

High-resolution X-ray crystallography reveals new details of conformational heterogeneity for the dynamic enzyme PTP1B.

## 1. Introduction

Proteins are dynamic molecules that undergo continuing motion. While it offers no insights into the timescales of such motions, X-ray crystallography can reveal detailed, atomistic information about the conformational ensemble of a protein. Such information can be represented in the form of multiconformer models with local alternate conformations (Keedy *et al.*, 2015 ▸; Riley *et al.*, 2021 ▸; Wankowicz *et al.*, 2023 ▸). The scale of the shifts between such alternate conformations can vary, ranging from small-scale backbone changes coupled to side-chain rotamer changes (Lovell *et al.*, 2000 ▸; Davis *et al.*, 2006 ▸) to larger-scale backbone shifts of secondary structure or loops (Deis *et al.*, 2014 ▸; Keedy *et al.*, 2018 ▸), although it is worth noting that even small-scale changes in protein conformation can be critical for biological function (Barstow *et al.*, 2008 ▸; Fraser *et al.*, 2009 ▸; Yabukarski *et al.*, 2020 ▸). Moreover, the local conformations of neighboring residues in proteins depend upon one another (Martin *et al.*, 2011 ▸; van den Bedem *et al.*, 2013 ▸; Bhattacharyya *et al.*, 2015 ▸; Johansson & Lindorff-Larsen, 2018 ▸). Importantly, even apo (unliganded) proteins are prone to sample low-occupancy conformations that change in population and contribute to function in response to molecular events (Keedy *et al.*, 2018 ▸; Wankowicz *et al.*, 2022 ▸; Greisman, Dalton *et al.*, 2023 ▸).

Despite the allure of X-ray crystallography for deciphering conformational heterogeneity in proteins, it has some technical limitations. Firstly, the resolution can be suboptimal, limiting our ability to resolve low-occupancy alternate conformations. Secondly, comparing two crystal structures from different experiments can be complicated by factors such as non-isomorphism, differences in crystallization conditions, cryocooling stochasticity (Keedy *et al.*, 2014 ▸; Fischer *et al.*, 2015 ▸) and coordinate error for small changes (Davis *et al.*, 2006 ▸). An ideal scenario would be a single crystal that diffracts to high resolution and reveals distinct protein states, thus allowing a controlled comparison between conformations and exploration of coupling between protein sites.

Here, we present a new crystal structure of the dynamic allosteric enzyme protein tyrosine phosphatase 1B (PTP1B; PTPN1; Keedy *et al.*, 2018 ▸; Whittier *et al.*, 2013 ▸; Choy *et al.*, 2017 ▸), the founding member (Tonks *et al.*, 1988 ▸) of the protein tyrosine phosphatases (PTPs). Our structure is the highest resolution (1.43 Å) structure of apo wild-type (WT) PTP1B to date out of a total of ∼350 structures of PTP1B deposited in the Protein Data Bank (PDB; Berman *et al.*, 2000 ▸), including mutant and ligand-bound structures. Furthermore, it assumes a rare crystal form displaying noncrystallographic symmetry, with two distinct copies of the protein in different environments within the same crystal lattice. This crystal form had never been observed for this protein until very recently in a series of structures bound to small-molecule fragments (Greisman, Willmore *et al.*, 2023 ▸; Morris *et al.*, 2023 ▸), but has not yet been reported for the apo enzyme and the conformational differences between the two distinct chains have not been studied. Another report included several structures of PTP1B with active-site mutations in the same space group, but they have different unit-cell dimensions, only one copy per asymmetric unit and lower resolution (Morris *et al.*, 2023 ▸), and thus are not directly relevant here. We exploit the fortuitous arrangement within our crystal to directly compare distinctly ordered states of PTP1B in atomic detail, including conformational differences that span distal regions of the structure such as key allosteric sites (Keedy *et al.*, 2018 ▸; Skaist Mehlman *et al.*, 2023 ▸).

We also compare our new structure with other notable, recently published structures of PTP1B. These include the recently published structures of WT PTP1B bound to small-molecule fragments at non-orthosteric sites in the same rare crystal form (Greisman, Willmore *et al.*, 2023 ▸). These liganded structures were not accompanied by an apo structure, which we now provide. Our analysis also includes mutant structures of PTP1B identified based on coevolution and designed to modulate dynamics (Torgeson *et al.*, 2022 ▸). These structures include the only structure of PTP1B at a higher resolution (1.24 Å) than ours (1.43 Å), but they do not include a structure of WT PTP1B and do not exhibit noncrystallographic symmetry. Our reanalysis of these published structures has unearthed additional ‘hidden’ conformational heterogeneity (Lang *et al.*, 2010 ▸) that was previously left unmodeled and helps to explain the functional effects of the mutations.

Overall, using our new high-resolution apo WT structure, we observe variable levels of conformational disorder in one protein chain versus another, with the effect being notably more pronounced for allosteric regions. We also highlight instances of coupled alternate conformations wherein one residue becomes more flexible while a neighboring residue becomes less flexible. Finally, we report a striking colocalization of (i) coupled alternate conformations in the apo state, (ii) activating mutations with surrounding residues exhibiting previously unmodeled structural responses and (iii) nearby small-molecule fragment binding. Together, our new data and the reanalysis of other recent data hint at an even broader allo­steric network in PTP1B than previously realized (Keedy *et al.*, 2018 ▸; Choy *et al.*, 2017 ▸) and highlight the value of high-resolution crystallography and multiconformer modeling for obtaining unique windows into protein conformational ensembles that may pertain to function.

## 2. Materials and methods

### 2.1. Protein expression and purification

For these experiments, PTP1B (residues 1–321) with an additional C-terminal His tag was expressed. Briefly, *Escherichia coli* BL21 cells were transformed with 10 ng His-tagged pET-21b-human PTP1B (1–321) plasmid and plated onto a lysogeny broth (LB) medium agar plate with ampicillin and incubated overnight at 37°C. A single bacterial colony was picked and grown overnight in LB medium with ampicillin at 37°C in a shaker incubator as the primary culture. The next day, the required amount of primary culture was added to fresh LB medium and allowed to grow at 37°C. Once the optical density (OD) reached 0.4–0.6, 1 m*M* ispropyl β-d-1-thiogalactopyranoside was added and the culture was grown overnight in a shaker incubator at 200°C. The culture was pelleted by centrifugation at 4000 rev min^−1^ for 1 h. The pellets were lysed immediately or stored at 20°C.

Bacterially expressed His-PTP1B (residues 1–321) was purified by nickel–nitrilotriacetic acid (Ni–NTA) affinity chromatography. Briefly, the bacterial pellet was solubilized in lysis buffer (20 m*M* NaH_2_PO_4_, 300 m*M* NaCl, 1 m*M* TCEP, protease-inhibitor cocktail tablet, pH 8) and lysed using a sonicator on ice (amplitude 40%, pulse on 5 s, pulse off 10 s, total 10 min). During sonication, an Ni–NTA column was equilibrated with lysis buffer. The lysate was centrifuged at 4000 rev min^−1^ and 40°C for 1 h and the supernatant was added to the pre-equilibrated Ni–NTA column and incubated for 1 h on a clinical rotor at 40°C. After incubation, the column was washed with five volumes of wash buffer (20 m*M* NaH_2_PO_4_, 300 m*M* NaCl, 1 m*M* TCEP, 20 m*M* imidazole pH 8) and finally eluted with elution buffer (20 m*M* NaH_2_PO_4_, 300 m*M* NaCl, 1 m*M* TCEP, 250 m*M* imidazole pH 8). Imidazole was removed from the protein using a Zeba Spin buffer-exchange column (ThermoFisher Scientific, model No. 89882) and PTP1B was stored in 5 m*M* TCEP solution (50 m*M* HEPES, 150 m*M* NaCl, 5 m*M* TCEP pH 8) at 40°C. Protein quantification was performed using the Bradford method. Purified PTP1B was then buffer-exchanged (50 m*M* HEPES, 150 m*M* NaCl pH 8) to remove TCEP before crystallization.

### 2.2. Crystallization

WT PTP1B at a stock concentration of 1 m*M* (final concentration of 0.30 m*M*) was incubated with water-soluble cholesterol at a stock concentration of 0.1 *M* (final concentration of 9.1 m*M*) in storage buffer (10 m*M* Tris pH 7.5, 0.2 m*M* EDTA, 25 m*M* NaCl, 3 m*M* DTT) for 3 h at room temperature.

This mixture was used to set up crystallization sitting drops in 96-well low-profile Art Robbins INTELLI-PLATE trays using an SPT Labtech Mosquito Xtal3 at a ratio of 0.1 µl protein solution to 0.1 µl well solution [0.1 *M* MgCl_2_, 0.1 *M* HEPES pH 7.0, 15%(*w*/*v*) PEG 4000; from the ProComplex commercial screen], which were then incubated at 4°C. Crystals grew within 5–7 days to a final size of 25–50 µm.

### 2.3. X-ray data collection

Crystals were harvested using MiTeGen microloops of appropriate size and then cryocooled by hand-plunging into liquid nitrogen. Diffraction data were collected remotely on the NYX beamline at the National Synchrotron Light Source II (NSLS-II). Single crystals were exposed to X-rays under a continuous cryostream (100 K), with 0.2° crystal rotation and 0.12 s exposure per image, for a total of 360° across 1800 images.

### 2.4. Crystallographic data processing and modeling

The diffraction data were processed using the *DIALS* pipeline (Winter *et al.*, 2022 ▸) via *xia*2 (Winter, 2010 ▸). All 1800 images (360°) were included in processing, and space group *P*4_3_2_1_2 was enforced. The resolution limit was automatically selected by *DIALS* (Winter *et al.*, 2022 ▸). The resulting data-processing statistics were favorable (Table 1 ▸).

The resulting merged structure-factor file was used for molecular replacement in *Phaser* (McCoy *et al.*, 2007 ▸) with PDB entry 1t49 as the search model. We also ran the program *xtriage* (Zwart *et al.*, 2005 ▸) to obtain the Matthews coefficient (Table 1 ▸). The Matthews coefficient of 2.21 Å^3^ Da^−1^ indicated the need to place two copies of the protein molecule in the asymmetric unit (non-identical copies).

Iterative modeling with *Coot* (Emsley *et al.*, 2010 ▸) was performed between rounds of refinement. Several iterations of refinement were performed using *phenix.refine* within the *Phenix* suite (Liebschner *et al.*, 2019 ▸). Refinement was performed with the ‘anisotropic *B* factor’ flag and ‘update waters’ set to true. ‘Update waters’ was turned off in later refinement rounds as the refinement approached convergence. By default, noncrystallographic symmetry between chains was not imposed. The resulting refinement statistics were favorable (Table 1 ▸).

### 2.5. Analysis of models

All structure figures were generated using *PyMOL* (Schrödinger).

The number of crystal contacts and total crystal surface area in Table 2 ▸ were calculated using the *PISA* web server (Krissinel & Henrick, 2007 ▸).

The average *B*-factor values for each chain in Table 2 ▸ were calculated in *Excel*.

Per-residue C^α^ distances between chains were calculated using *VMD* (Humphrey *et al.*, 1996 ▸) using the following steps: 
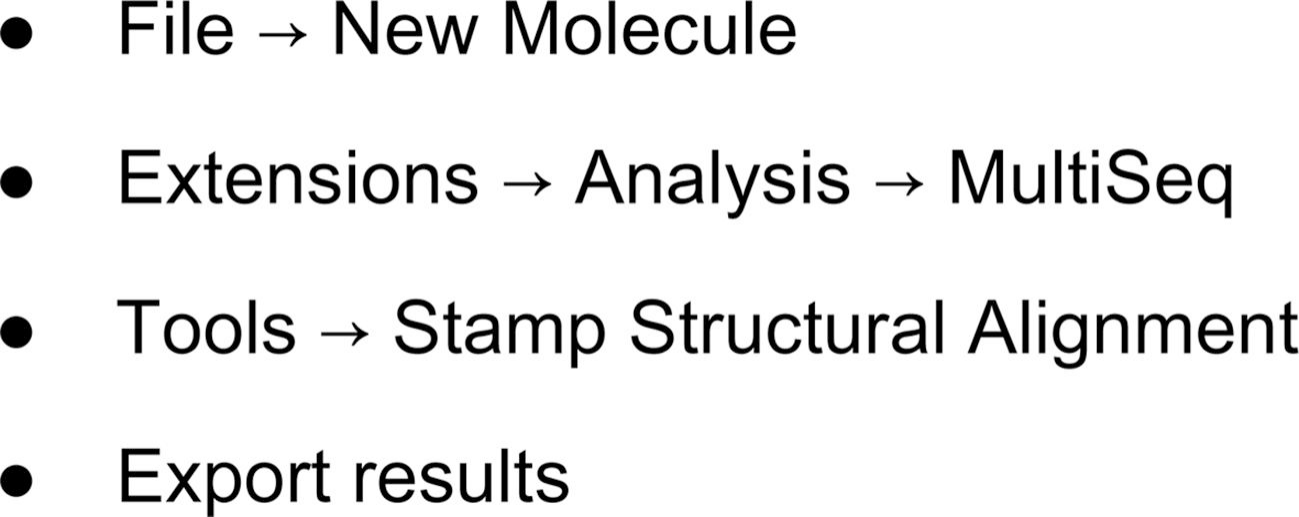


We use the ‘alignto’ command in *PyMOL* to overlay structures using chain *A* of each structure as a reference in Supplementary Fig. S3, PDB entry 8u1e chain *A* as a reference in Supplementary Fig. S8 and PDB entry 8u1e chain *B* as a reference in Supplementary Fig. S9.

Numbers of alternate conformations and of water molecules were calculated using custom awk one-line scripts.

The spectrum bars representing the ranges of C^α^-distance and *B*-factor values were created using the spectrumbar.py script from PyMOL Wiki. The colors used for the spectrum bar are (in order) blue, cyan, green and yellow, with rectangular ends, a radius of 1.5 and a length of 50.0.

Coloring the structures by C^α^ distance was performed using the colorbyrmsd.py script from PyMOL Wiki.

The PTP1B pseudo-ensemble was constructed using the advanced search feature at the PDB website with a threshold of 95% sequence identity to the sequence of PDB entry 1sug. All 351 structures were included in the rotamer analysis. The *phenix.rotalyze* utility was used to calculate all rotamers per residue, which were then compared across all chains of all structures. We calculated the average occurrence of all rotamers for all residues in our structure compared with the rest of the structures in the pseudo-ensemble.

*qFit* was run using the deposited model and structure-factor files for PDB entry 8u1e. Alternate conformations (except conformer *A*) were deleted and the structure was refined (*phenix.refine*). A composite omit map was then created using the refined model and the original structure factors (*phenix.composite_omit_map*). Finally, *qFit* was run using the refined model and composite omit map (qfit_protein composite_omit_map -l labels refined_model) followed by iterative *qFit* refinement (qfit_final_refine_xray.sh structure_factors.mtz qfit_output.pdb).

For the openness of the L16 site, *ProDy* (Bakan *et al.*, 2011 ▸, 2014 ▸) was used to calculate the distance between the C^α^ atom of Met235 and the C^α^ atom of Lys239 for all chains of all PTP1B structures available in the PDB.

For the pocket volume of the L16 site, *CASTp* (*Computed Atlas of Surface Topography of proteins*; Tian *et al.*, 2018 ▸) was used to calculate the pocket volume of the L16 site for chain *A* and chain *B*, both chains for isomorphous structures in the same space group, and PTP1B structures with small-molecule fragments bound in the L16 site. The L16 site was visually matched using the *CASTp* interactive feature.

## 3. Results

### 3.1. Unique crystallographic data set for PTP1B

We have determined a new crystal structure of apo WT PTP1B to high resolution (1.43 Å; Table 1 ▸). Our structure is the highest resolution apo structure of WT PTP1B to date; the second highest resolution for a deposited apo structure of WT PTP1B is 1.50 Å (PDB entry 2cm2; Ala *et al.*, 2006 ▸) and the third highest is 1.70 Å (Supplementary Fig. S1). Although data-reduction methodologies and the metrics for choosing resolution cutoffs have evolved over time, our structure is still certainly among the top two highest resolution apo WT structures of PTP1B. Moreover, the space group of our data set is *P*4_3_2_1_2, which is rare for PTP1B. Indeed, among the ∼350 crystal structures of PTP1B deposited in the PDB, spanning seven different space groups, only five other deposited structures have the same crystal form (with the same unit-cell dimensions) as our structure, albeit all with bound ligands, grown in a condition different to ours and from one recent study (Greisman, Willmore *et al.*, 2023 ▸). Thus, the structure we report here is the first of apo PTP1B in this new crystal form. Although *R*_gap_ (*R*_free_ − *R*_work_) for our structure is 5.2%, which might be considered somewhat high, some previous structures in the same space group, which were determined independently by other scientists, have similarly high or higher *R*_gap_ values, including several in the same crystal form (Supplementary Fig. S2).

Notably, this unusual crystal form of PTP1B includes two non-identical protein chains in the asymmetric unit. In the following sections, we interrogate differences in conformational heterogeneity between these two chains in detail, with an eye toward potential involvement in allosteric mechanisms.

### 3.2. Global structural differences between chains

Numerous studies using X-ray crystallographic data have established the role of crystal packing/contacts on the observed conformational landscape of a protein (Jacobson *et al.*, 2002 ▸; Bhabha *et al.*, 2015 ▸; Tyka *et al.*, 2011 ▸). Our new structure of PTP1B contains two nonidentical chains in the asymmetric unit, each experiencing distinct packing within the crystal lattice. In particular, chain *B* has more crystal contacts and a greater total crystal contact surface area than chain *A* (Table 2 ▸). Consistent with the idea that more extensive crystal packing can stabilize protein conformations whereas less extensive packing can allow conformational disorder, chain *B* has lower *B* factors than chain *A*, whether considering C^α^ backbone atoms or all atoms including side chains (Table 2 ▸). These changes in protein disorder appear to be correlated to significant changes in solvation at the protein surface: strikingly, chain *B* has 49% more ordered water molecules than chain *A* (Table 2 ▸). Together, these observations illustrate how the two distinct chains in our new high-resolution structure provide a useful avenue to explore how local conformational changes, in this case from crystal contacts, can affect other parts of a protein structure.

**Figure 1:**
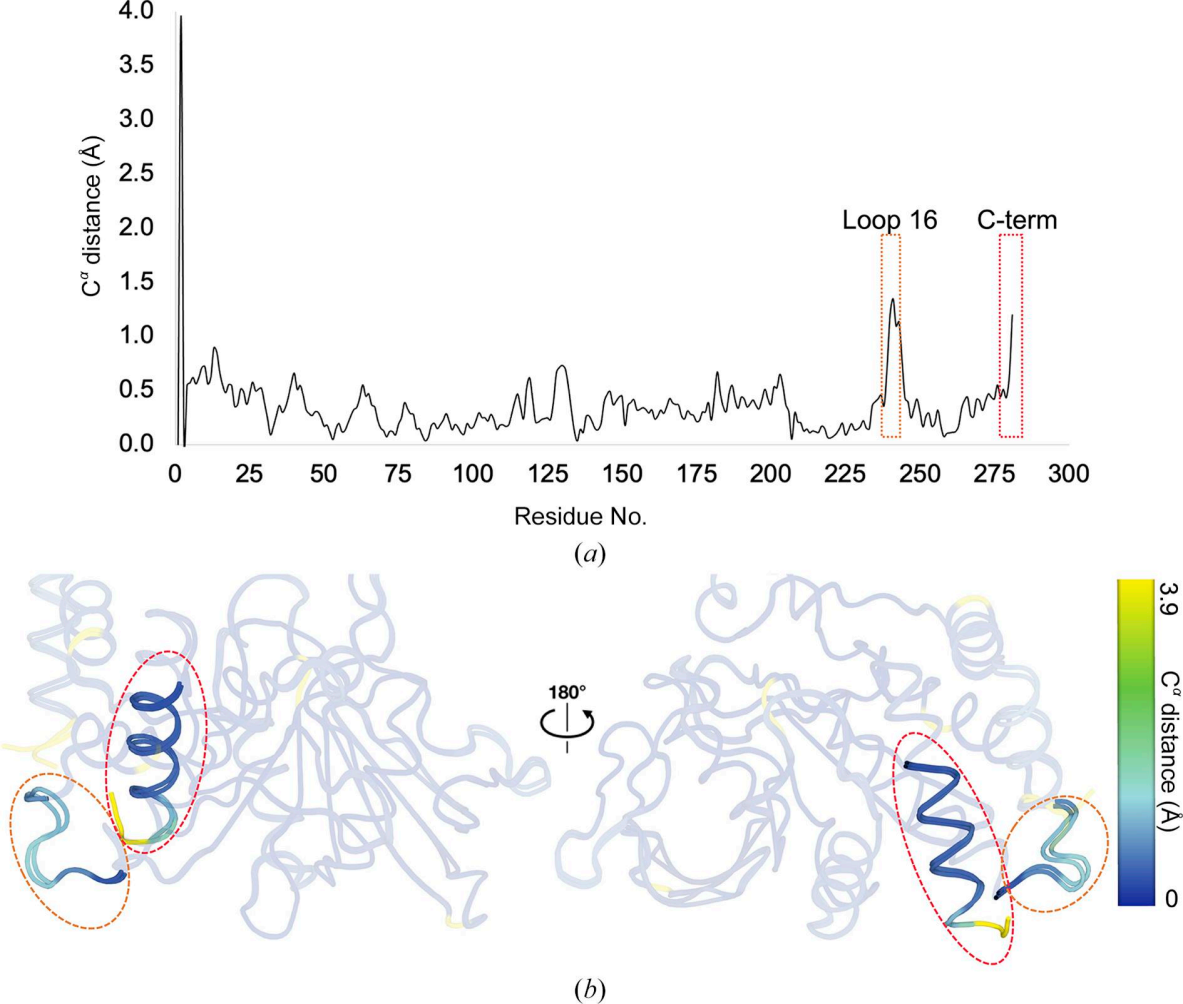
Sites displaying high backbone displacement between two distinct protein chains. (*a*) Plot of inter-chain C^α^ distance versus amino-acid sequence. (*b*) Overlay of both chains with residues colored by inter-chain C^α^ distance, viewed from two different angles. Regions of interest in (*a*) and (*b*) are highlighted with colored dashed outlines.

The next part of our analysis focuses on specific regions with backbone movements between the two chains, based on the C^α^–C^α^ distance after superposition. Interestingly, we observe that the residues with the largest differences across the two chains generally correspond to regions previously annotated as being allosteric, including loop 16 of the L16 allosteric site, the N-terminus adjacent to the L16 site, and the C-terminus including the allosteric α7 helix (Fig. 1 ▸). Several active-site loops including the WPD loop maintain a relatively similar conformation between chains.

We also performed the same analysis using the only other five structures of PTP1B in the same crystal form (all ligand-bound; PDB entries 8g6a, 8g65, 8g67, 8g68 and 8g69; Greisman, Willmore *et al.*, 2023 ▸). The results point to the same allosteric regions as in our new structure, albeit with even greater effects in some regions, such as the allosteric L16 site and the C-terminus in PDB entry 8g69 (Supplementary Fig. S3). The results for the ligand-bound structures also point to some additional areas, including the active-site E loop (residues ∼110–120) and the region near residues 60–65, both of which can be difficult to model into local density and exhibit coordinate variability across the various PTP1B structures in the PDB. More globally, pairwise C^α^ r.m.s.d. analysis shows that each chain is self-similar across the series of isomorphous structures. For example, across all pairs of these structures, the r.m.s.d. ranges from 0.12 to 0.31 Å when comparing chain *A* of one structure with chain *A* of another structure, and from 0.12 to 0.28 Å when comparing chain *B* with chain *B*; by contrast, the r.m.s.d. ranges from 0.27 to 0.43 Å when comparing chain *A* with chain *B*.

### 3.3. Differences in disorder from *B* factors

**Figure 2:**
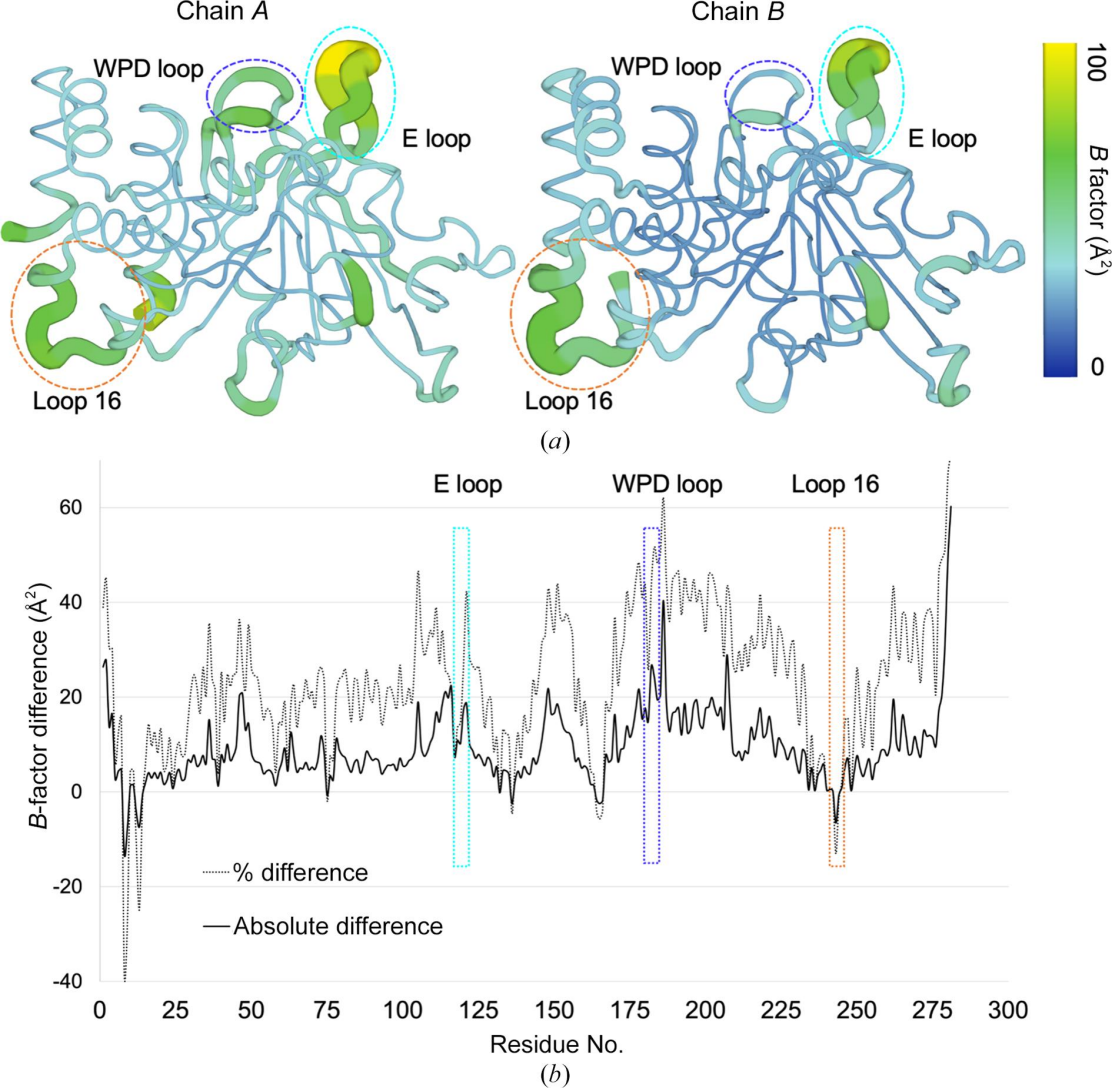
Differences in atomic disorder across sites and between distinct protein chains. (*a*) All-atom *B* factors in chain *A* (left) versus chain *B* (right). (*b*) All-atom absolute (solid line) and relative (%, dotted line) *B*-factor difference (chain *A* minus chain *B*) plotted as a function of amino-acid sequence. In both panels, notable regions with high disorder and/or differential disorder between the two chains are highlighted with dashed lines (blue, WPD loop; cyan, E loop; pink, L16 allosteric site).

To test whether overall differences in disorder between the two chains (Table 2 ▸) are distributed evenly versus heterogeneously throughout the protein structure, we studied *B* factors on a per-residue basis. As the two chains are from the same crystal structure, no extra normalization of *B* factors is required. A side-by-side comparison of the *B* factors in the two chains of our structure implicates the active-site WPD loop, active-site E loop and allosteric L16 site as exhibiting relatively high disorder (Fig. 2 ▸ *a*). *B*-factor differences between chains indicate differential conformational effects in these regions: for example, the WPD loop is more flexible in chain *A*, whereas the L16 site is more flexible in chain *B* (Fig. 2 ▸ *b*). Thus, despite chain *A* being more flexible overall, different local regions are more or less flexible in either chain.

### 3.4. Local structural differences between chains

**Figure 3:**
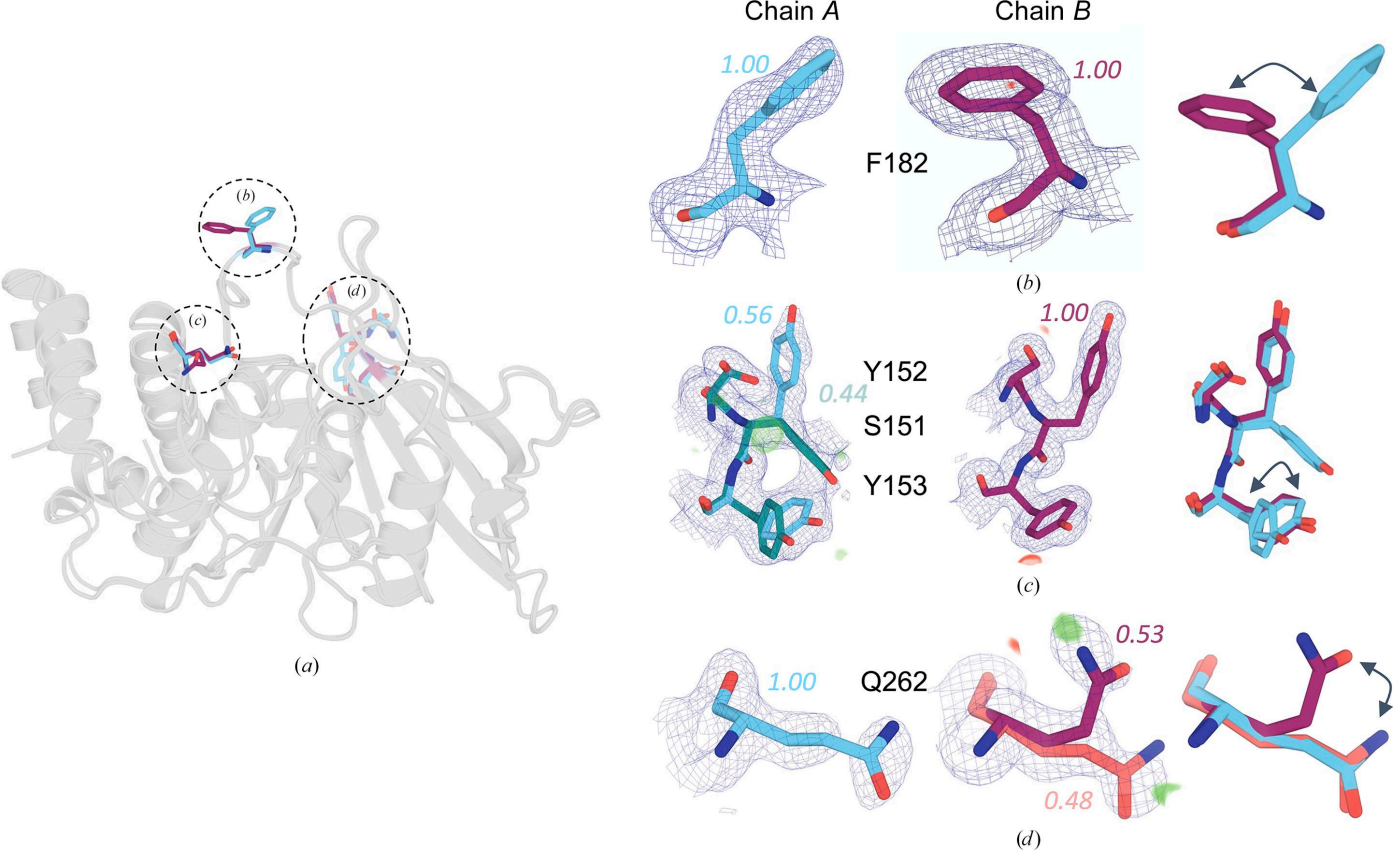
Examples of inter-chain differences in local alternate conformations. These examples demonstrate the differences in side-chain conformations between chains *A* (cyan) and *B* (maroon). (*a*) Overlay of chains *A* and *B*, highlighting the locations of the residues displayed in (*b*)–(*d*). (*b*) Phe182 of the WPD loop adopts a different conformation in each chain (2*F*_o_ − *F*_c_ map in blue mesh, 1σ). (*c*) The allosteric loop 11 (Keedy *et al.*, 2018 ▸) is flexible in chain *A* but is more ordered in chain *B* (2*F*_o_ − *F*_c_ map in blue mesh, 0.5σ; *F*_o_ − *F*_c_ map shown as green/red volume, ±3σ; alternate conformation *A* in cyan and *B* in teal). (*d*) Gln262 of the active-site Q loop adopts a single conformation in chain *A* but alternate conformations in chain *B* (2*F*_o_ − *F*_c_ map in blue mesh, 0.75σ; *F*_o_ − *F*_c_ map shown as green/red volume, ±3σ; alternate conformation *A* in maroon and *B* in salmon).

A superposition of the two chains in our new structure reveals several differences in local conformation (Fig. 3 ▸ *a*). Firstly, although the WPD loop is in the open conformation in both chains of our structure, only in chain *B* does Phe182 (the F in the WPDFG motif; Yeh *et al.*, 2023 ▸) adopt a side-chain rotamer conformation that is rare for PTP1B, occurring in only ∼2% of crystal structures (χ_1_ **t** near 180°; Lovell *et al.*, 2000 ▸; Fig. 3 ▸ *b*, middle panel; Supplementary Fig. S4).

Secondly, a set of correlated alternate conformations can also be seen in loop 11 (L11; residues 151–153). The conformations of residues in this loop have previously been shown to be correlated to the WPD loop and α7 helix (Keedy *et al.*, 2018 ▸). In our structure, chain *A* adopts dual conformations for these residues (Fig. 3 ▸ *c*, left panel), whereas chain *B* adopts a single conformation (Fig. 3 ▸ *c*, middle panel).

Thirdly, another residue from the active site, Gln262 in the Q loop, which mediates hydrolysis of the phosphocysteine intermediate as part of the catalytic mechanism (Brandão *et al.*, 2010 ▸), samples two conformations only in chain *B* (Fig. 3 ▸ *d*, middle panel). This is in contrast to chain *A*, where Gln262 can only be modeled in a single conformation (Fig. 3 ▸ *d*, left panel).

For Phe182, the conformational difference can be attributed to a unique direct crystal contact in chain *B*. In loop 11, the situation is similar, although the direct crystal contact only involves residues 151–152 from the loop, suggesting that the inter-chain differences for the other residues in the loop (Fig. 3 ▸ *c*) may be due to conformational coupling in this allosteric region (Keedy *et al.*, 2018 ▸). These local instances of crystal contacts in chain *B* but not in chain *A* are consistent with chain *B* exhibiting more crystal contacts and lower protein flexibility overall (Table 2 ▸). In contrast to these first two examples, Gln262 is remote from any crystal contacts in either chain. The alternate conformation of Gln262 is somewhat rare for PTP1B and is observed in only 19% of all PTP1B structures (Supplementary Fig. S4). Overall, only seven out of 28 residues with alternate conformations in chain *A* and four out of 29 in chain *B* are apparently affected by crystal contacts. These observations indicate that most conformational differences between chains in our structure arise not from direct crystal contacts but rather from indirect or allosteric effects from nonlocal crystal contacts. Additionally, although we demonstrate inter-chain differences using only three regions (Fig. 3 ▸), a further rotamer analysis across the two chains shows that 24% of all residues display different rotamer(s), accounting for pairwise comparisons between alternate conformations.

To assess the extent to which the manually modeled alternate conformations in our structure are recapitulated by an automated algorithm, we also used *qFit* (Riley *et al.*, 2021 ▸; Wankowicz *et al.*, 2023 ▸). Our alternate conformations are matched by *qFit* for many residues (Supplementary Fig. S5) but not for others (Supplementary Fig. S6). Many of the cases without a side-chain match involve protein backbone motions; *qFit* does model backbone motions, but has room for improvement in this area. Overall, despite a comparable number of alternate conformations across the two chains, *qFit* recapitulates more of our alternate conformations in the more well ordered chain *B* for several different definitions of matching conformations (Supplementary Table S1).

Most of the rotamers in our structure occur with appreciable frequency in a pseudo-ensemble of all PTP1B structures from the PDB (see Section 2; Supplementary Fig. S4). However, 54 residues (19%) in chain *A* and 54 residues (19%) in chain *B* have rarer rotamers that are seen in fewer than 20% of the PDB structures; this includes the interesting rotamers highlighted in Fig. 3 ▸ (Supplementary Fig. S4).

### 3.5. Diverse conformations of an allosteric region near the C-terminus

**Figure 4:**
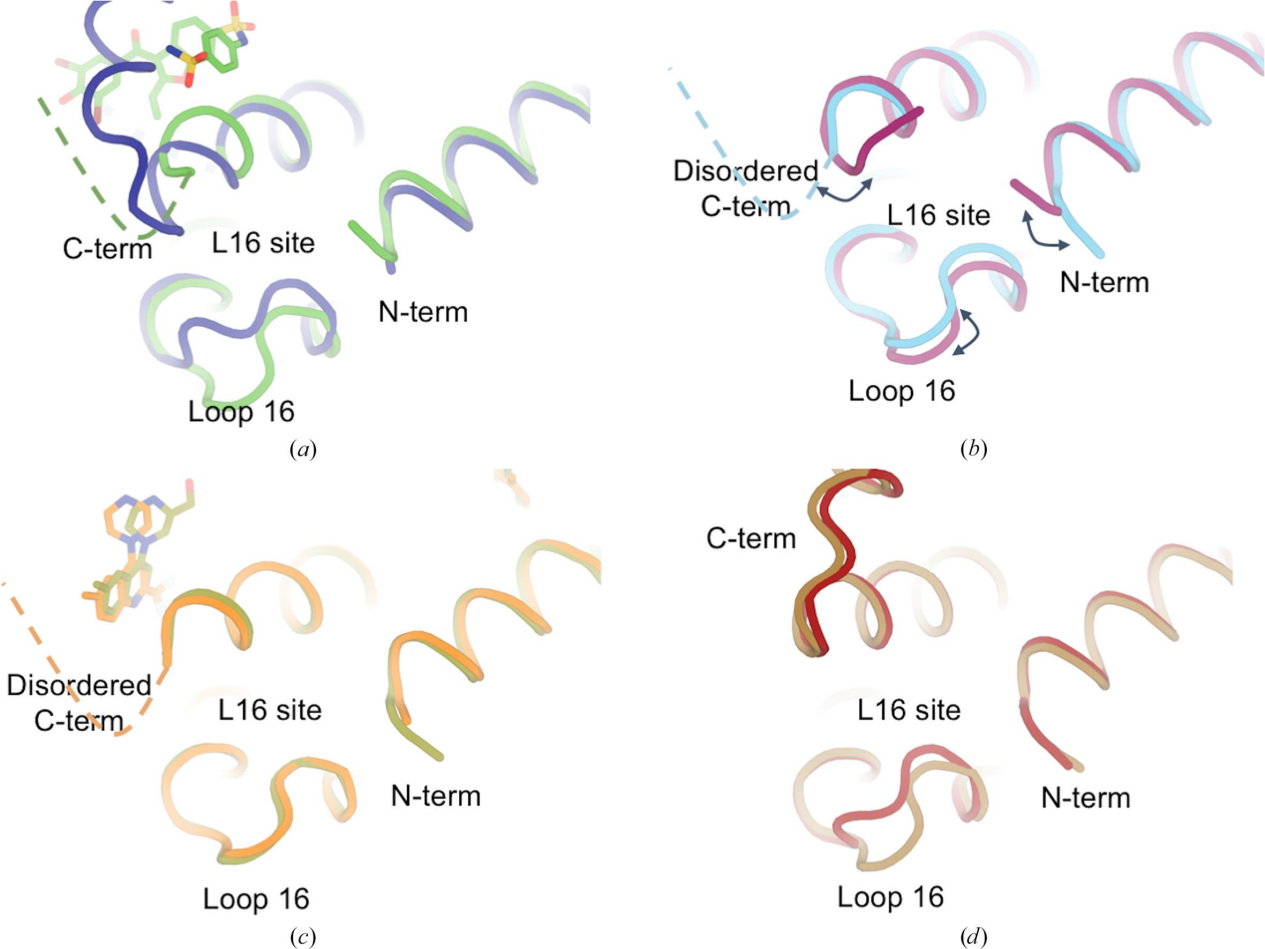
Unexpected differences in the allosteric L16 site involving loop 16, the N-terminus and the C-terminus. (*a*) Comparison of PTP1B in the closed state (PDB entry 1sug, dark blue) and in the open state bound to an allosteric inhibitor at the nearby BB site (PDB entry 1t49, green) shows large changes in the regions constituting the allosteric L16 site. (*b*) An overlay of the two chains in our new structure (cyan, chain *A*; maroon, chain *B*) shows more subtle but significant differences in the three regions of the L16 site. (*c*) Two structures in the *P*4_3_2_1_2 ligand-bound series (PDB entry 8g6a chain *A*, olive; PDB entry 8g67 chain *A*, orange; Greisman, Willmore *et al.*, 2023 ▸) show little to no change in the three regions. (*d*) Two other structures in the *P*4_3_2_1_2 ligand-bound series (PDB entry 8g69 chain *A*, dark red; PDB entry 8g65 chain *A*, brown; Greisman, Willmore *et al.*, 2023 ▸) show a uniquely reordered C-terminus as well as changes in loop 16.

Of the ∼350 structures of PTP1B in the PDB, the majority exhibit a disordered and therefore unmodeled C-terminus. The ordering of the C-terminus is shown to be loosely coupled to the conformation of the WPD loop and the allosteric L16 site (Keedy *et al.*, 2018 ▸; Sharma *et al.*, 2023 ▸; Skaist Mehlman *et al.*, 2023 ▸). In a previously published closed-state structure of PTP1B, the C-terminus was modeled as ordered with the L16 site in the closed conformation (Fig. 4 ▸ *a*; Pedersen *et al.*, 2004 ▸). This can be contrasted with an open-state structure of PTP1B bound to an allosteric inhibitor, in which the C-terminus is disordered and the L16 site is open (Fig. 4*a*; Wiesmann *et al.*, 2004 ▸).

In our new high-resolution structure, a closer inspection of these regions shows disorder in the C-terminus in both chains, and a difference between chains in the L16 site involving a partial opening in chain *B* (∼1.4 Å C^α^ shift), coupled to a differently ordered N-terminus (Fig. 4 ▸ *b*). The L16 site shift is notable because this loop typically exhibits bistable behavior, toggling between discrete open or closed states, but here exhibits an extra-open state (Supplementary Fig. S7*b*). More generally, the changes seen between chains in our structure are notable in that they are smaller than, but reminiscent of, the changes observed between the canonical open and closed states of PTP1B (Fig. 4 ▸ *a*).

A similar comparison with the recently reported ligand-bound *P*4_3_2_1_2 structures indicates disorder in the C-terminus when two different small-molecule fragments bind in the nearby BB allosteric site (Fig. 4 ▸ *c*), similar to what was observed with the BB3 allosteric inhibitor (Fig. 4 ▸ *a*). In addition, in some other structures in this crystal form with other small-molecule fragments bound elsewhere at a seemingly unrelated location in PTP1B, the C-terminus shows dramatic reordering into a nonhelical conformation (Fig. 4 ▸ *d*), in this case stabilized by extensive crystal contacts in only one of the two chains. Consistent with C^α^-distance analysis (Supplementary Figs. S3, S8 and S9), L16 is modeled in either the open or the closed state in different structures (Fig. 4 ▸ *d*). This is somewhat surprising given the previously reported correlation between an ordered C-terminal α7 helix and a closed L16 site (Keedy *et al.*, 2018 ▸); it is possible that differently ordered C-terminus conformations have different allo­steric effects on nearby regions. More generally, these observations are consistent with a previous report that the C-terminal α7 helix region can reorder into significantly distinct conformations, albeit involving adjacent bound ligands (Keedy *et al.*, 2018 ▸), further suggesting that this key region of PTP1B is quite conformationally malleable.

The various conformations of the L16 site also provide insights into the ligandability of the site. Firstly, we applied a distance metric for L16 site openness to the entire PTP1B pseudo-ensemble, including both chains of our new structure and the isomorphous liganded structures. This analysis showed that most structures in the new crystal form are unremarkable with regard to this site, yet a few are relatively unusual (Supplementary Figs. S7*a* and S7*b*). Specifically, the L16 site in chain *B* of our new structure is more open than most open-state structures in the pseudo-ensemble and is the most open of all structures in our unique crystal form (Supplementary Figs. S7*b* and S7*c*). By contrast, chain *A* of one of the isomorphous structures (PDB entry 8g69) is among the most closed for L16, approaching an intermediate-like state (Supplementary Fig. S7*a*).

Secondly, we analyzed the L16 site pocket volume for a subset of PTP1B structures. The results showed that both chains of our new structure had minimal pocket volume (Supplementary Fig. S10), but several other isomorphous structures, each with an unliganded L16 site, had similar pocket volumes as structures with small-molecule fragments bound in the L16 site, indicating that these conformations may provide ligandable but subtly different targets for the design of structure-based allosteric modulators.

### 3.6. Unmodeled alternate conformations for activating mutations

**Figure 5:**
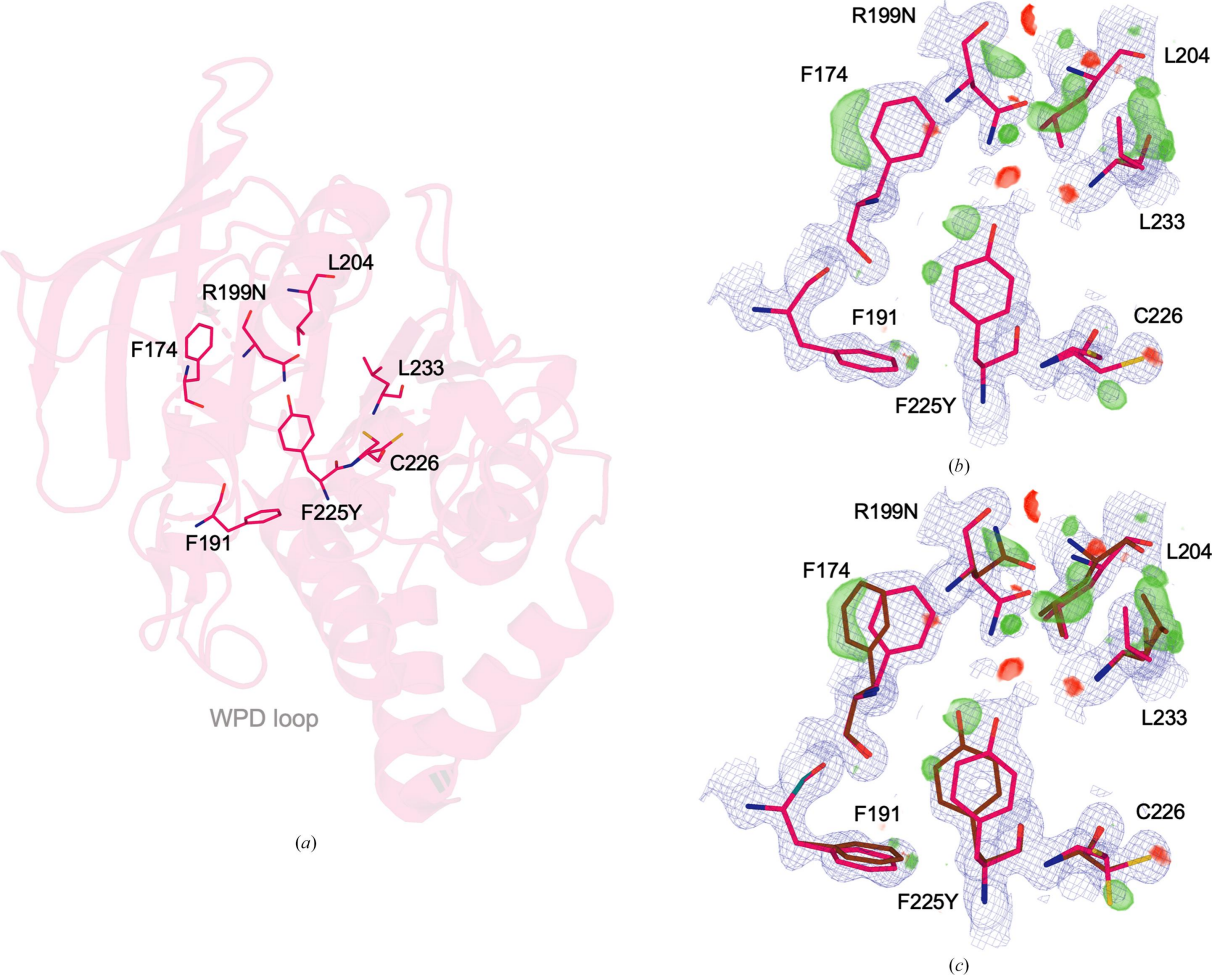
Modeling missing alternate conformations for a high-resolution double-mutant structure. A cluster of residues centered around and including the point mutations F225Y and R199N in a high-resolution (1.24 Å) double-mutant structure of PTP1B (PDB entry 7mn9; Torgeson *et al.*, 2022 ▸) exhibits difference electron-density features suggestive of unmodeled alternate conformations. Original conformations are in pink; manually modeled alternate conformations are in brown. (*a*) Zoomed-out view of the residues displayed in subsequent panels. (*b*) Original model from PDB entry 7mn9 (2*F*_o_ − *F*_c_ map in blue mesh, 1σ; *F*_o_ − *F*_c_ map in green/red meshes, ±3σ). (*c*) Updated model with manually modeled missing alternate conformations (2*F*_o_ − *F*_c_ map in blue mesh, 1σ; *F*_o_ − *F*_c_ map in green/red meshes, ±3σ).

Prior mutational analysis has provided information about the intramolecular interaction network in PTP1B, with certain sets of residues exhibiting evidence of coupling (Choy *et al.*, 2017 ▸; Hjortness *et al.*, 2018 ▸; Torgeson *et al.*, 2022 ▸). One such analysis (Torgeson *et al.*, 2022 ▸) demonstrated how double point mutations identified by co-evolutionary sequence analysis (F225Y–R199N), distal from the active site, enhance catalysis by and reduce the stability of PTP1B. Upon closer inspection of the high-resolution F225Y–R199N crystal structure in the regions around the double mutations (Fig. 5 ▸), we observe evidence in the form of difference electron-density patterns for unmodeled alternate conformations of several relevant residues, including both of the mutated residues themselves (F225Y and R199N) as well as several surrounding residues (including, but not limited to, Phe191, Phe174, Leu233, Leu204 and Cys226; Fig. 5 ▸ *b*). Upon modeling the missing conformations, including distinct side-chain rotamers, subtler side-chain shifts within rotameric wells and more complex movements of backbone plus side chains that are less straightforward to model, it becomes evident that these sites are more conformationally heterogeneous than originally modeled (Fig. 5 ▸ *c*). Of particular note among these residues, Phe174 helps to form the ‘floor’ of the 197 allosteric site (Keedy *et al.*, 2018 ▸) and Phe191 interacts directly with Tyr179 of the active-site WPD loop. Notably, these residues (Phe225, Arg199, Phe174, Leu204 and Leu233) show no evidence of alternate conformations in either chain of our WT structure, suggesting that the mutations increase dynamics throughout this local cluster, although the differing resolutions of the two structures (1.24 versus 1.43 Å) may complicate this interpretation. Taken together, our new observations here are consistent with the hypothesis based on NMR experiments that increased dynamics for the double mutant give rise to the increased activity and reduced stability (Torgeson *et al.*, 2022 ▸) and provide atomistic insights into the conformational states that may be involved in these dynamics.

### 3.7. Correlated conformations cluster near sites of mutations and ligands

**Figure 6:**
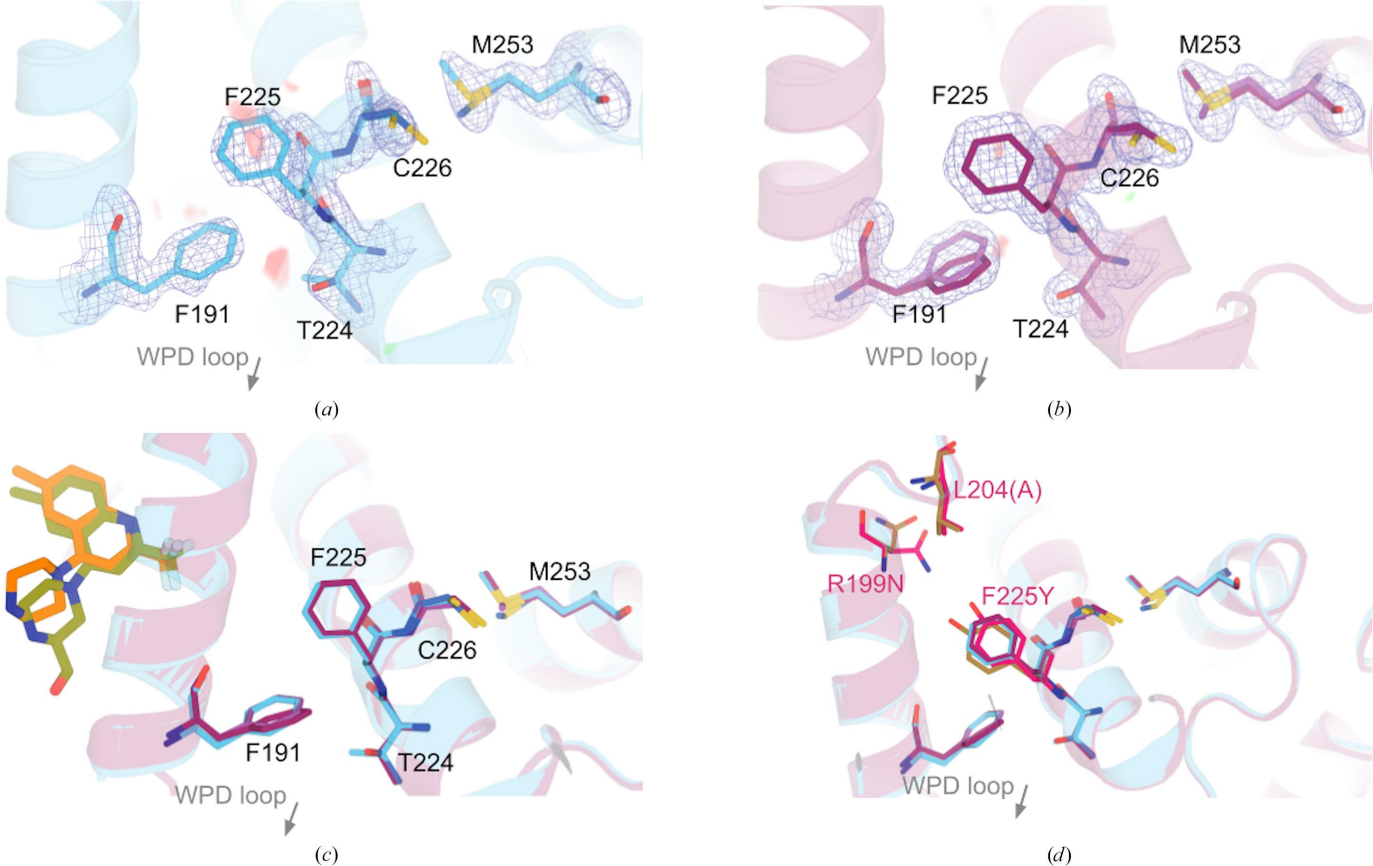
Clustered, correlated conformations localize to functionally linked and/or ligandable sites. Two sub-clusters of alternate conformations are identified, with one around the BB site (Wiesmann *et al.*, 2004 ▸) and the other not previously reported in the allosteric network. Since these two sub-clusters are bridged by the functionally linked Phe225 (Torgeson *et al.*, 2022 ▸), they cohere as a single coupled cluster. (*a*, *b*) Our new high-resolution apo WT structure (chain *A*, cyan; chain *B*, maroon) and electron density (2*F*_o_ − *F*_c_ map in blue mesh, 1σ). We observe two adjacent sub-clusters with correlated alternate conformations. One involves the BB pathway (Phe191) and the other has not previously been reported as allosteric. (*c*) One of the two sub-clusters is also adjacent to recently reported small-molecule fragments that bind in the adjacent BB allosteric site, from the only structure series in the same crystal form as our structure (PDB entry 8g6a chain *A*, olive; PDB entry 8g67 chain *A*, orange; Greisman, Willmore *et al.*, 2023 ▸). (*d*) The two sub-clusters are also adjacent to several residues shown to influence activity upon mutation, including Phe225 which bridges the two sub-clusters (Torgeson *et al.*, 2022 ▸). Here, we show an overlay of the F225Y–R199N double-mutant structure (pink, PDB entry 7mn9) with our WT structure. Additional remodeled conformations that were originally missing (see Fig. 5 ▸) are colored brown.

Correlated motions between residues may convey allosteric information between distal sites in proteins such as PTP1B (Choy *et al.*, 2017 ▸). We explored our multiconformer structure for any sites that may undergo such correlated motions, and identified two neighboring ‘sub-clusters’ (Figs. 6 ▸ *a* and 6 ▸ *b*). Residues Phe191 and Thr224 in these sub-clusters seem to exhibit compensating conformational heterogeneity, such that alternate conformations for one residue coincide with a single conformation for the other residue in one chain, with the situation reversed in the other chain.

Interestingly, these sub-clusters lie adjacent to three regions of interest. The first is the catalytic WPD loop, including the eponymous Trp179 ‘anchor’, which directly contacts Phe191, a residue which samples alternate conformations in one chain of our new structure (Fig. 6 ▸ *b*). The second is the known BB allosteric site (Wiesmann *et al.*, 2004 ▸; Greisman, Willmore *et al.*, 2023 ▸; Fig. 6 ▸ *c*). The third is a site of several recently reported activating point mutations (Torgeson *et al.*, 2022 ▸; Fig. 6 ▸ *d*). These mutations include F225Y, which exhibits (previously unmodeled) conformational heterogeneity in the F225Y–R199N double mutant (Fig. 5 ▸). Thus, Phe225 serves as a ‘bridge’ between the two sub-clusters that we observe in the WT enzyme, which can thus be considered to form one cohesive cluster. This cluster collectively exhibits a combination of existing dynamics in WT PTP1B as well as the capacity for altered dynamics upon local perturbations that are likely to impact enzyme function.

## 4. Discussion

In this study, we present the highest resolution structure to date of apo WT PTP1B. This high resolution affords a detailed view of the conformational ensemble of this enzyme. Our structure is also the first of apo PTP1B in the recently discovered *P*4_3_2_1_2 crystal form. For context, out of a total of 350 structures of PTP1B in the PDB, the representation of different crystal forms is far from uniform: there are seven unique space groups, but 80% of structures are in space group *P*3_1_21. Based on our new apo structure as well as five other recent isomorphous ligand-bound structures (Greisman, Willmore *et al.*, 2023 ▸), the distinct packing in this crystal form appears to favor high-resolution diffraction, thereby helping to provide new windows into the conformational landscape of PTP1B.

Unlike most other PTP1B structures, the unusual crystal form of our new structure also accommodates two copies of the protein in distinct crystal-packing environments. The distinct patterns of crystal contacts experienced by these two chains within the same asymmetric unit (Table 2 ▸) allowed us to perform controlled comparisons of the conformational ensemble of PTP1B without the need to scale structure factors across data sets (Aldama *et al.*, 2023 ▸) or normalize *B* factors across models (Ringe & Petsko, 1986 ▸; Carugo & Argos, 1997 ▸; Vihinen *et al.*, 1994 ▸). Notably, the chain that was least impacted by crystal contacts exhibited noticeably higher disorder, with visually dispersed electron density, higher *B* factors (Table 2 ▸, Fig. 2 ▸) and more alternate conformations in several regions (Fig. 3 ▸). Detailed inter-chain comparisons revealed that functional sites, such as the active site and allosteric sites, had the largest responses in terms of backbone shifts (Figs. 1 ▸ and 4 ▸), side-chain rotamer changes (Fig. 3 ▸) and atomic disorder monitored by *B* factors (Fig. 2 ▸). This local­ization of conformational flexibility to functional sites is consistent with the view that protein dynamics underlies and enables biological function (Tzeng & Kalodimos, 2009 ▸; Fraser *et al.*, 2009 ▸; Wei *et al.*, 2016 ▸; Kim *et al.*, 2017 ▸; Greisman, Dalton *et al.*, 2023 ▸).

In particular for PTP1B, we highlight a region centered on the α4 helix, including the flanking α3 and α5 helices, that exhibits coupled conformational heterogeneity. Interestingly, this region lies adjacent to the previously identified BB allosteric site (Wiesmann *et al.*, 2004 ▸), which also binds recently reported small-molecule fragments (Greisman, Willmore *et al.*, 2023 ▸; Fig. 6 ▸ *d*). Notably, several mutations in this area have recently been reported to affect enzyme function, including F225Y and R199N, which together increase catalysis and decrease stability (Torgeson *et al.*, 2022 ▸). Our reanalysis of the previously reported high-resolution structure of the F225Y–R199N double mutant reveals compelling evidence in the electron density for missing, unmodeled alternate conformations of these two mutated residues as well as several neighboring residues (Fig. 5 ▸), complementing our observations of conformational heterogeneity in this region of our new high-resolution WT structure. These crystallographic observations thus serve as an unexpected additional validation of the previously proposed idea, based on NMR data and assuming a rigid crystal structure, that these mutations influence PTP1B function by imparting changes in protein dynamics (Torgeson *et al.*, 2022 ▸). These results are also consistent with the notion that WT PTP1B may harbor latent dynamic wiring that can be modulated by mutations to alter function (Tokuriki & Tawfik, 2009 ▸).

The alternate conformations of the double mutant mentioned above were especially identifiable due to the particularly high resolution of the structure: indeed, it has the highest resolution of all 350 available PTP1B structures (1.24 Å). However, unmodeled alternate conformations are surprisingly common in crystal structures across the PDB (Lang *et al.*, 2010 ▸; Riley *et al.*, 2021 ▸; Wankowicz *et al.*, 2023 ▸; Stachowski & Fischer, 2023 ▸). If properly modeled, they could likely help to explain the functional effects of mutations and ligands (Wankowicz *et al.*, 2022 ▸), allosteric mechanisms and other phenomena for PTP1B and other systems. Moreover, other biophysical perturbations (Keedy, 2019 ▸), such as variable temperature (Fraser *et al.*, 2011 ▸; Keedy *et al.*, 2014 ▸, 2018 ▸; Fischer, 2021 ▸; Stachowski *et al.*, 2022 ▸; Sharma *et al.*, 2023 ▸; Skaist Mehlman *et al.*, 2023 ▸; Greisman, Dalton *et al.*, 2023 ▸; Thorne, 2023 ▸) or pressure (Urayama *et al.*, 2002 ▸; Guerrero *et al.*, 2023 ▸), could reveal additional, previously ‘hidden’ conformational heterogeneity or excited states that could provide further insights into the biological mechanisms of PTPs as well as other proteins. These areas represent promising avenues for future study.

## 5. Data availability

Model, structure-factor and map files are available in the Protein Data Bank under PDB accession code 8u1e. Raw X-ray diffraction images for this data set have also been deposited in the SBGrid Data Bank and are available at https://doi.org/10.15785/SBGRID/1060.

## Supplementary Material

PDB reference: PTP1B, 8u1e

Raw X-ray diffraction images.: https://doi.org/10.15785/SBGRID/1060

Supplementary Table and Figures. DOI: 10.1107/S2053230X23010749/jt5072sup1.pdf

## Figures and Tables

**Table 1:** Crystallographic statistics Values in parentheses are for the highest resolution.

PDB code	8u1e
Resolution (Å)	30.59–1.43 (1.48–1.43)
Completeness (%)	97.94 (85.28)
Multiplicity	25.6 (25.7)
〈*I*/σ(*I*)〉	10.82 (0.31)
*R*_merge_(*I*)	0.147 (9.777)
*R*_meas_(*I*)	0.150 (9.974)
*R*_p.i.m._(*I*)	0.029 (1.955)
CC_1/2_	1.000 (0.404)
Wilson *B* factor (Å^2^)	24.84
Total observations	3052767 (302442)
Unique observations	119377 (11763)
Space group	*P*4_3_2_1_2
*a*, *b*, *c* (Å)	88.41, 88.41, 163.00
α, β, γ (°)	90, 90, 90
Solvent content (%)	44.80
*R*_work_	0.150 (0.352)
*R*_free_	0.202 (0.392)
R.m.s.d., bond lengths (Å)	0.010
R.m.s.d., angles (°)	1.04
Ramachandran outliers (%)	0.36
Ramachandran favored (%)	97.33
Clashscore	3.35
*MolProbity* score	1.25

**Table 2:** Differences in structural metrics between chains See Section 2 for calculation details.

	Chain *A*	Chain *B*	Difference (%)
No. of crystal contacts	3	5	+67
Total crystal contact surface area (Å^2^)	749.7	1869.7	+149
Average *B* factor, C^α^ atoms (Å^2^)	35.5	26.7	−25
Average *B* factor, all atoms (Å^2^)	38.6	29.5	−24
No. of ordered water molecules	156	233	+49
