# Supplementary material for: High-resolution double vision of the allosteric phosphatase PTP1B
Source: Acta Crystallogr F Struct Biol Commun. 2024 Jan 1;80(Pt 1):1–12. doi: 10.1107/S2053230X23010749 (PMC10833341; doi:10.1107/S2053230X23010749)
Supplement: Supplementary file 1 [file f-80-00001-sup1.pdf]

Acta Cryst F STRUCTURAL BIOLOGY COMMUNICATIONS

Volume 80 (2024)

Supporting information for article:

High-resolution double vision of the allosteric phosphatase PTP1B

Shivani Sharma, Tamar (Skaist) Mehlman, Reddy Sudheer Sagabala, Benoit Boivin and Daniel A. Keedy

Table S1 Cross-chain comparison of rotamers for residues with alternate conformations in 8u1e vs. qFit model.

	Chain A	Chain B
Residues with alt confs in original model	29	28
Residues with alt confs in qFit model	52	79
Residues with simple match in qFit model *	31.0%	67.9%
Residues with $\chi 1$ match in qFit model **	17.2%	57.1%
Residues with exact match in qFit model ***	6.9%	46.4%

The different parameters used for each comparison are detailed below and marked with asterisks.

* simple match = For each residue with alt confs in the original model, the residue in the qFit model has alt confs, regardless of rotamer(s).

** $\chi 1$ match = For each alt-conf rotamer in the original model, the qFit model residue has a rotamer with the same basin (e.g., p/t/m (Lovell et al. 2000)) for the $\chi 1$ dihedral angle.

*** exact match = For each alt-conf rotamer in the original model, the residue in the qFit model has a rotamer that matches exactly; for example, tttt and tttt would match, but tttt and tttm would not.

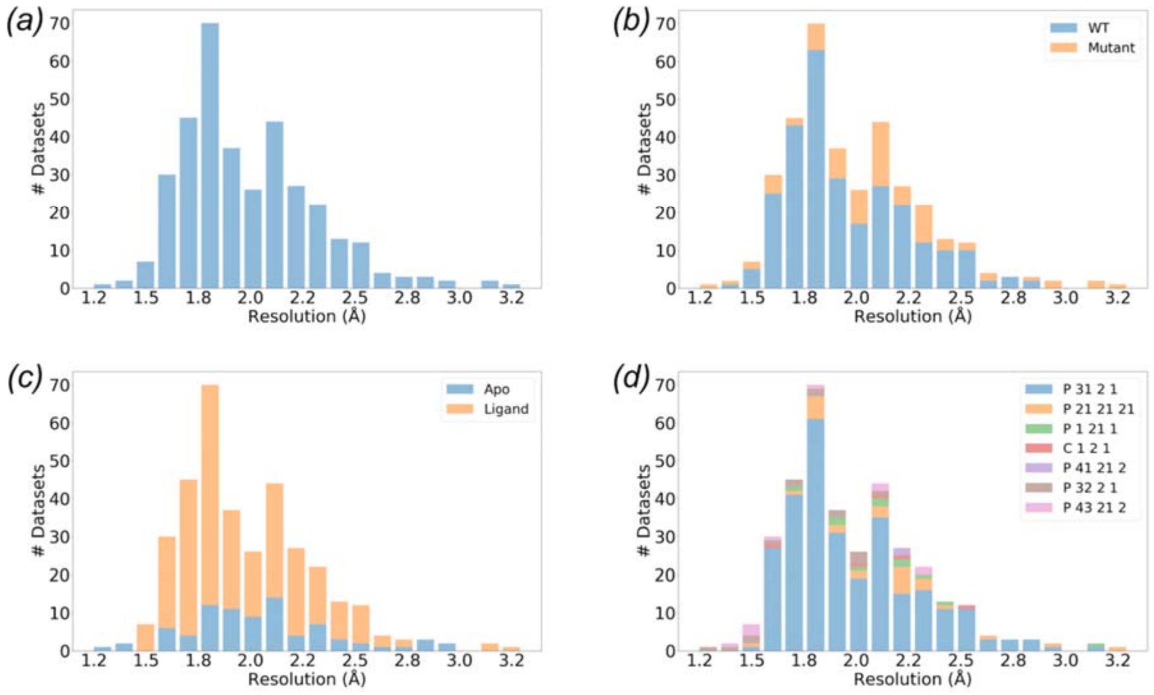

Figure S1 Resolutions of PTP1B structures in the PDB. Histogram of 351 PTP1B structures' resolutions separated by category. *(a)* Overview of all structures. *(b)* Colored by WT vs. mutant. *(c)* Colored by apo vs. ligand-bound. *(d)* Colored by space group.

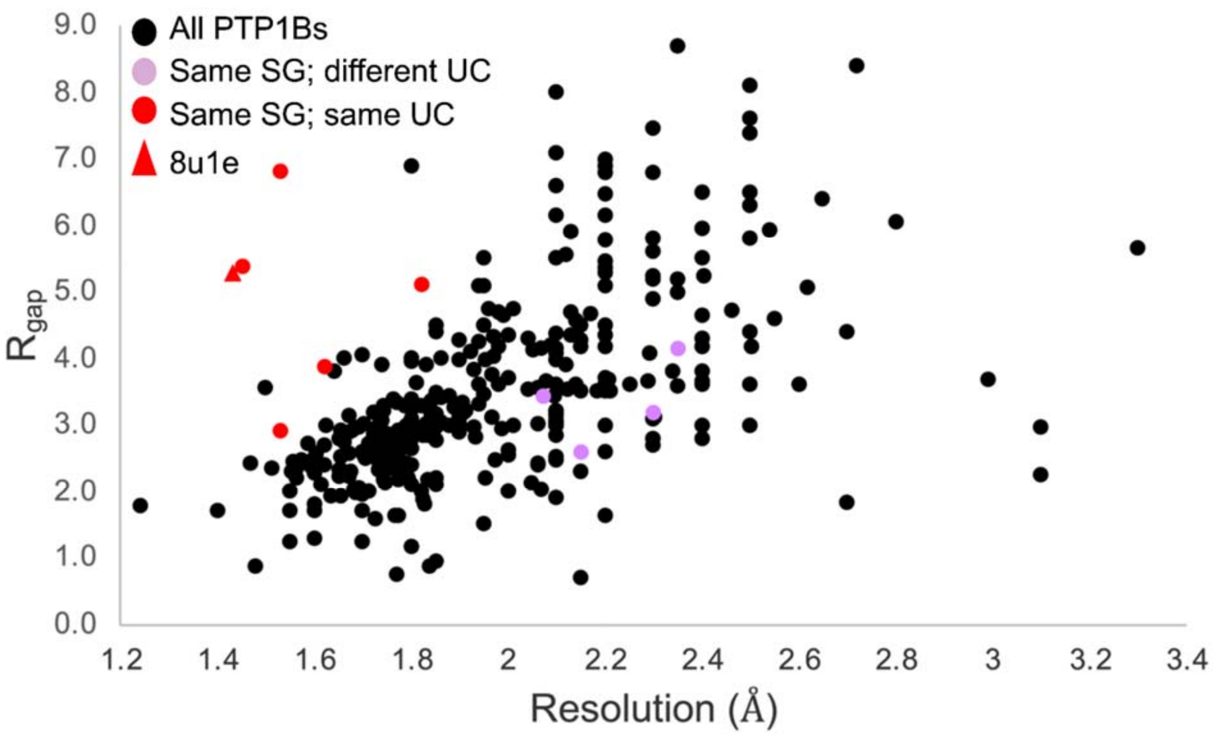

Figure S2 Distribution of R_{gap} values for all PTP1B structures. R_{gap} (R_{free} - R_{work}) R_{gap} is plotted against resolution for all PTP1B structures in the PDB. R_{gap} values are similar for our structure (red triangle) compared to other isomorphous P 43 21 2 structures (red circles) at similar resolutions.

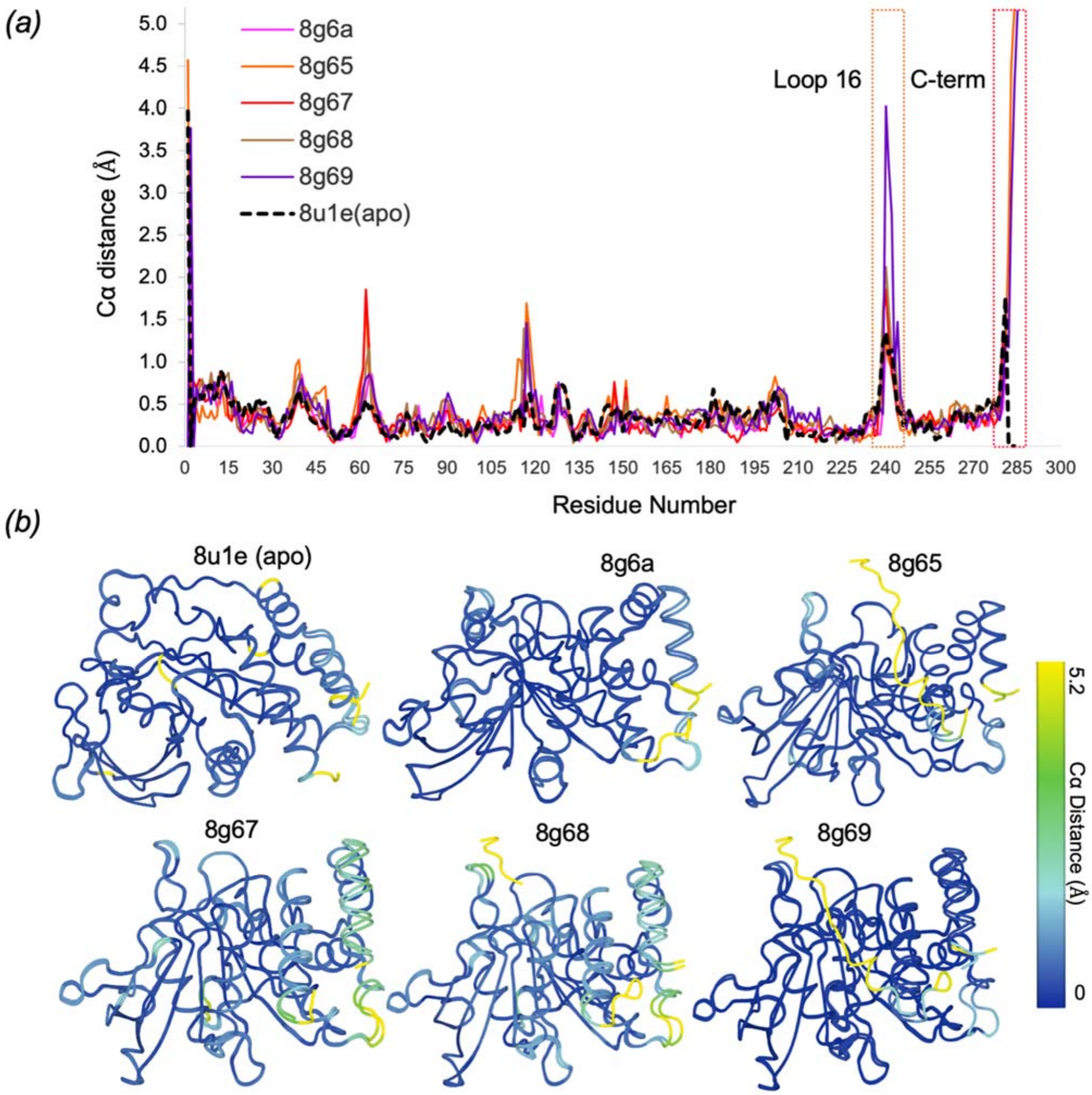

Figure S3 Backbone displacements between chains across the P 43 21 2 series. Regions with highest backbone variability between the two chains are highlighted with colored dotted outlines for our high-resolution apo structure and the isomorphous ligand-bound structure series (Greisman, Willmore, et al. 2023). *(a)* Plot of inter-chain Cα distance vs. amino acid sequence. *(b)* Overlay of both chains with residues colored by inter-chain Cα distance.

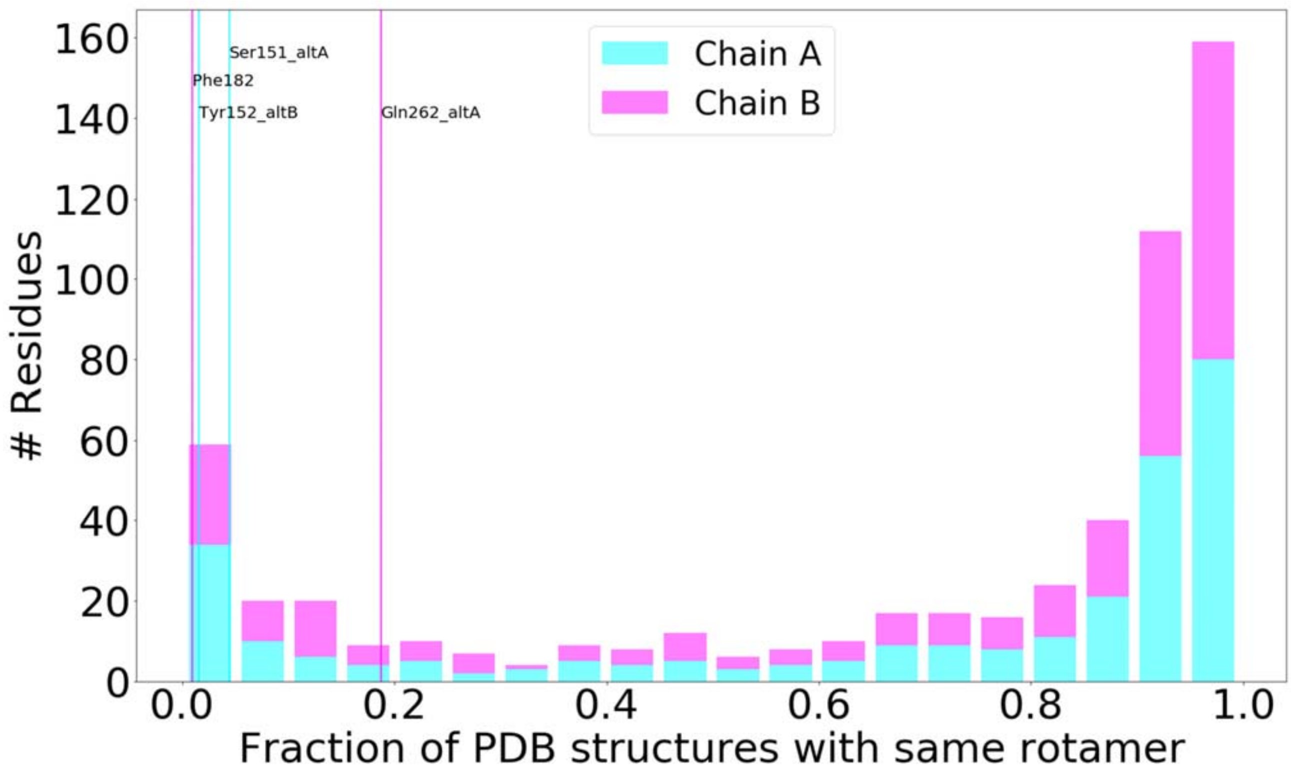

Figure S4 Rarity of rotamers compared to the PDB. Stacked histogram illustrating the fraction of PTP1B structures in the PDB with the same rotamer for chain A (cyan) and chain B (maroon).

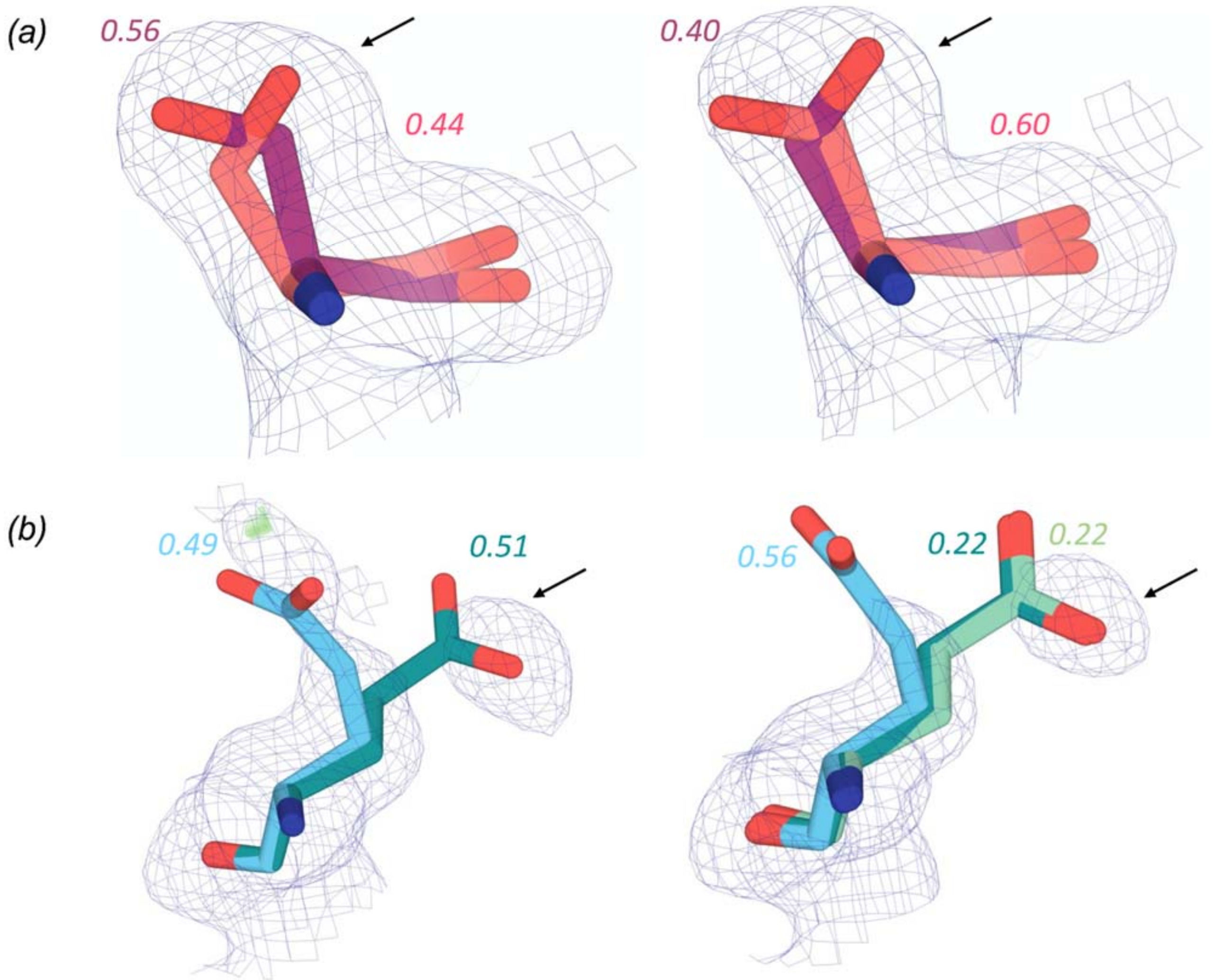

Figure S5 Example residues in 8u1e with alternate conformations that are recapitulated by qFit. For each panel, our original deposited model (PDB ID: 8u1e) is on the left, and the qFit model is on the right. Occupancies are labeled to match the color of the conformation. Arrows indicate density peaks that help justify the placement of the alternate conformations. Final refined density is shown for the original structure; a composite omit map, used as input for qFit, is shown for the qFit model (contoured at same level as 2Fo-Fc). *(a)* Ser28, chain B. Alternate conformation A in maroon, B in salmon. 2Fo-Fc as blue mesh, 1 σ; Fo-Fc as green/red volume, +/-3 σ. *(b)* Glu75, chain A. Alternate conformation A in cyan, B in teal, and C in pale green. 2Fo-Fc as blue mesh, 0.5 σ; Fo-Fc as green/red volume, +/-3 σ.

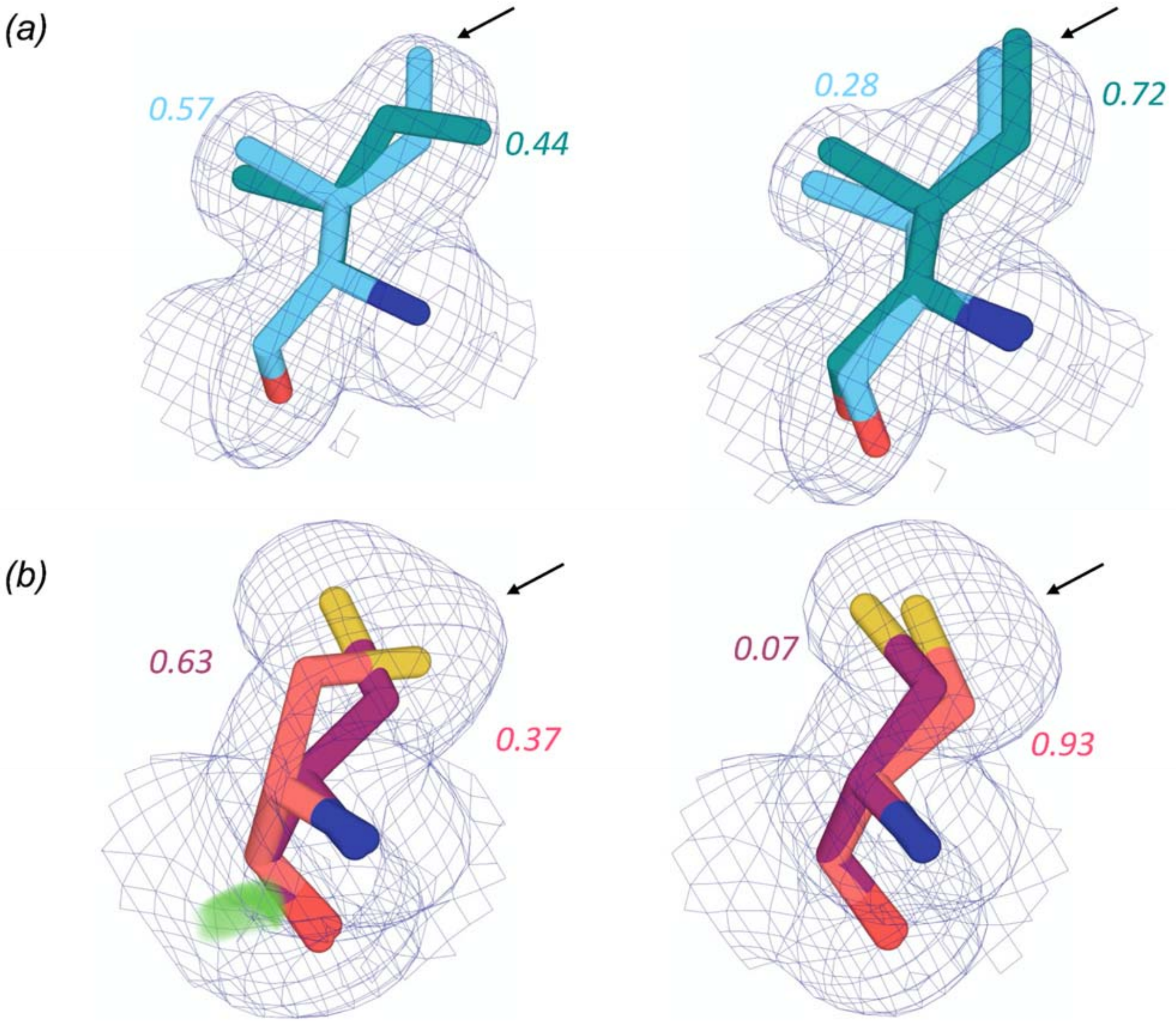

Figure S6 Example residues in 8u1e with alternate conformations that are not recapitulated by qFit. For each panel, our original deposited model (PDB ID: 8u1e) is on the left, and the qFit model is on the right. Occupancies are labeled to match the color of the conformation. Arrows indicate density peaks that help justify the placement of the alternate conformations. Final refined density is shown for the original structure; a composite omit map, used as input for qFit, is shown for the qFit model (contoured at same level as 2Fo-Fc). *(a)* Ile72, chain A. Alternate conformation A in cyan, B in teal). 2Fo-Fc as blue mesh, 1 σ; Fo-Fc as green/red volume, +/- 3 σ. *(b)* Cys121, chain B. Alternate conformation A in maroon, B in salmon. 2Fo-Fc as blue mesh, 1 σ; Fo-Fc as green/red volume, +/- 3 σ.

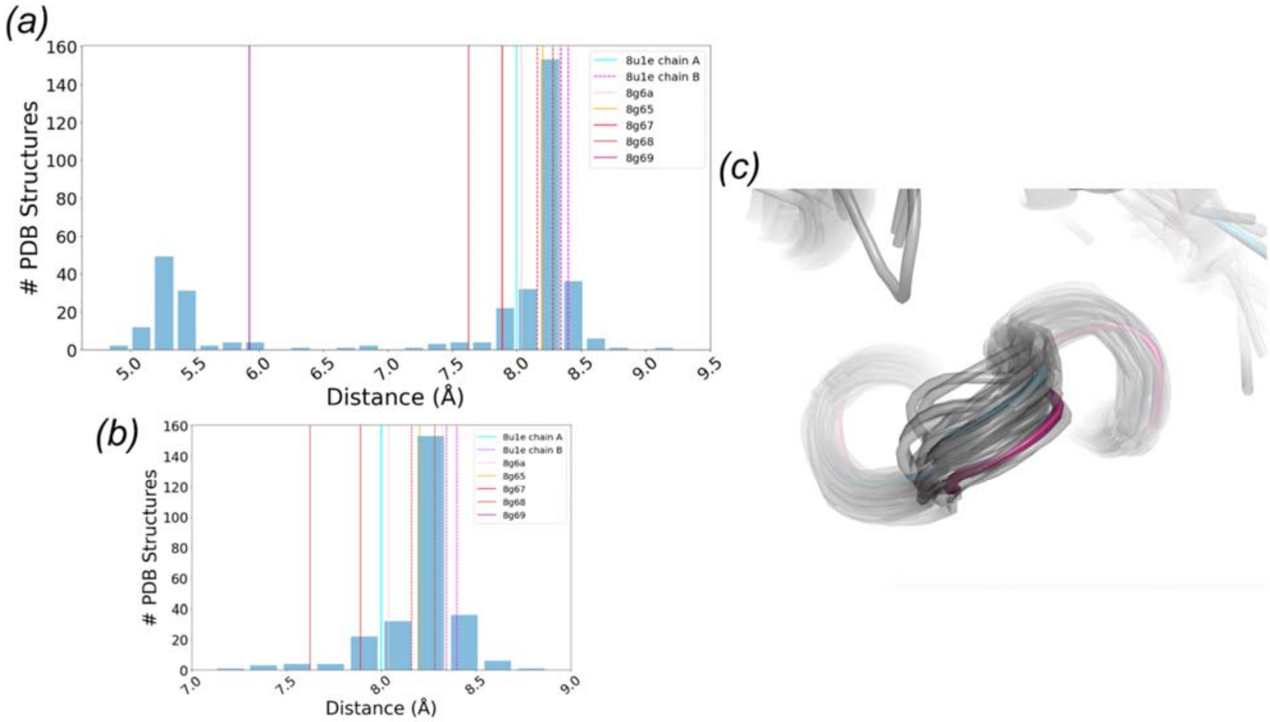

Figure S7 L16 site openness for all PTP1B structures. The L16 site is seen across all structures of PTP1B to be either closed or open, but to varying degrees. *(a)* Histogram of the Cα–Cα distance between Met235 and Lys239 for the 351 PTP1B structures in the PDB. The distances for the P 43 21 2 structures are shown with vertical lines, with chain A in solid lines and chain B in dashed lines. In most, the site is open, but to different degrees across the chains within the same PDB, and in one chain the site is closed. *(b)* Zoom-in of the histogram region associated with an open L16 site. *(c)* Overlay of all PDB structures with an open L16 site. Chain A (cyan) and B (maroon) from our structure are highlighted; both are open but chain B is more open than most.

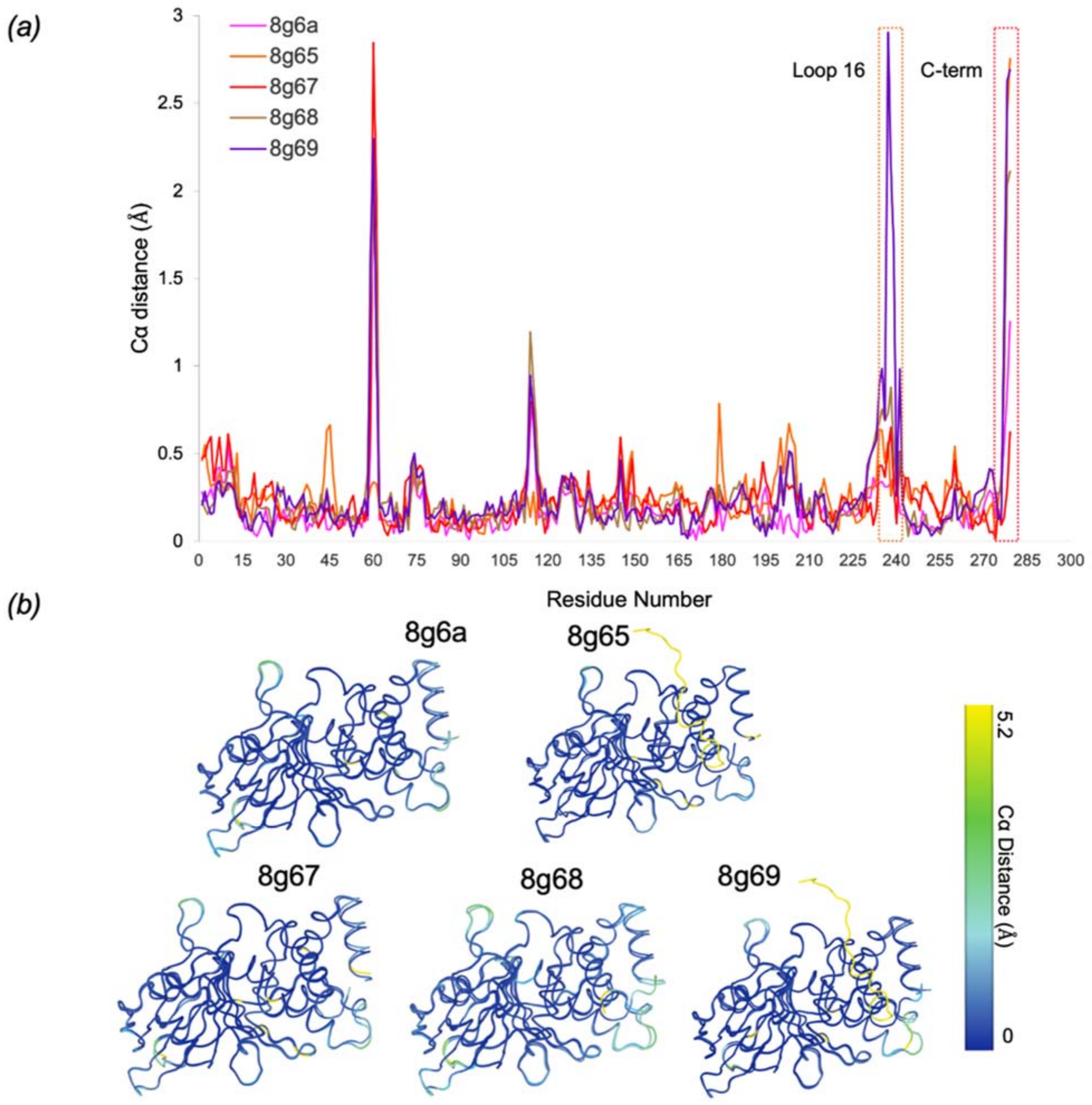

Figure S8 Backbone displacements between chain A of 8u1e (apo) and of the ligand-bound P 43 21 2 series. Regions with highest backbone variability between structures for chain A are highlighted with colored dotted outlines for our high-resolution apo structure and the isomorphous ligand-bound structure series (Greisman, Willmore, et al. 2023). *(a)* Plot of inter-structure (8u1e as reference) Cα distance vs. amino acid sequence. *(b)* Overlay of chain A in 8u1e and the compared structure with residues colored by Cα distance.

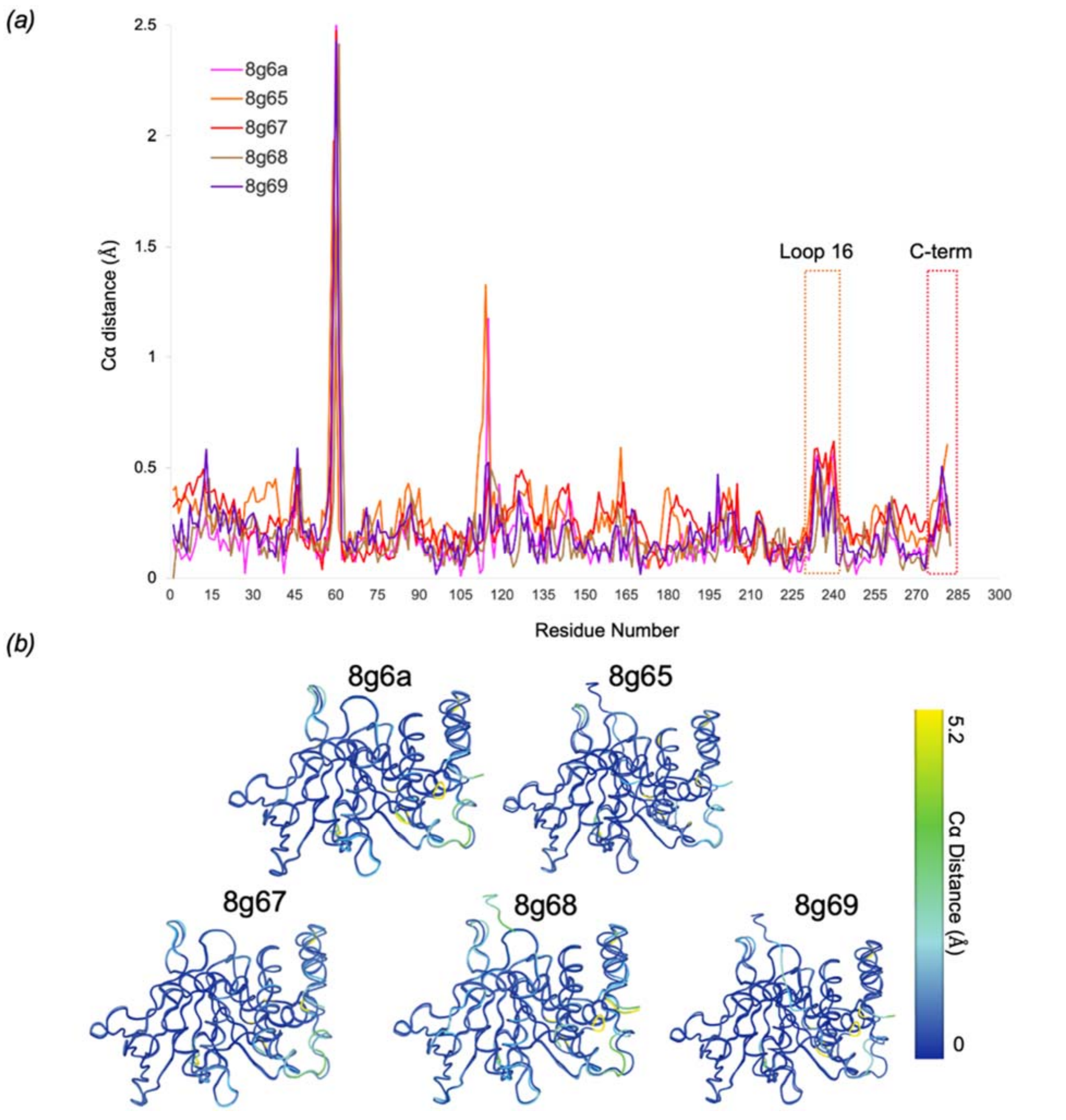

Figure S9 Backbone displacements between chain B of 8u1e (apo) and of the ligand-bound P 43 21 2 series. Regions with highest backbone variability between structures for chain B are highlighted with colored dotted outlines for our high-resolution apo structure and the isomorphous ligand-bound structure series (Greisman, Willmore, et al. 2023). *(a)* Plot of inter-structure (8u1e as reference) Cα distance vs. amino acid sequence. *(b)* Overlay of chain B in 8u1e and the compared structure with residues colored by Cα distance.

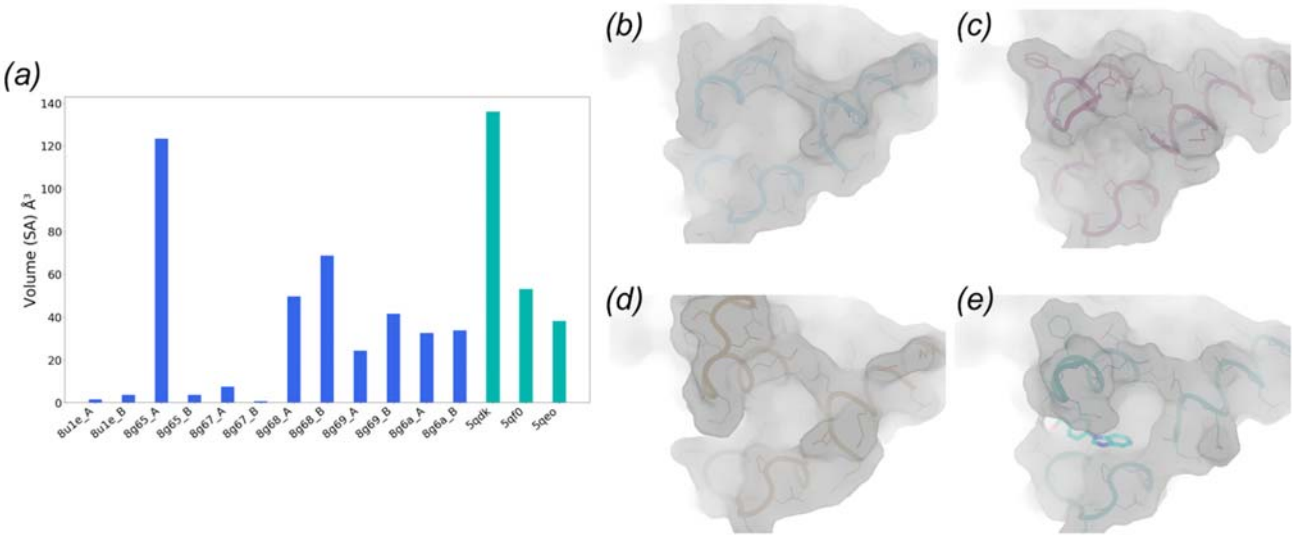

Figure S10 Comparison of L16 pocket volume. The L16 pocket volume varies across chains of the P 43 21 2 structures as well as ligand bond structures, as seen in surface representation of the L16 pocket for select examples. *(a)* Volume of the L16 pocket in the P 43 21 2 structures (blue) and ligand-bound structures (green). *(b)* Surface representation of our structure, chain A. *(c)* Surface representation of our structure, chain B. *(d)* Surface representation of 8g65, chain A. *(e)* Surface representation of the fragment-bound structure 5qdk.
